# Early detection of colorectal cancer by leveraging Dutch primary care consultation notes with free text embeddings

**DOI:** 10.1038/s41598-023-37397-2

**Published:** 2023-07-04

**Authors:** Torec T. Luik, Ameen Abu-Hanna, Henk C. P. M. van Weert, Martijn C. Schut

**Affiliations:** 1 Department of Medical Informatics, Amsterdam Public Health Research Institute, Amsterdam UMC, University of Amsterdam, Amsterdam, The Netherlands; 2 Department of General Practice/Family Medicine, Amsterdam Public Health Research Institute, Amsterdam UMC, University of Amsterdam, Amsterdam, The Netherlands

**Keywords:** Risk factors, Cancer, Computer science, Scientific data

## Abstract

We aimed to assess the added predictive performance that free-text Dutch consultation notes provide in detecting colorectal cancer in primary care, in comparison to currently used models. We developed, evaluated and compared three prediction models for colorectal cancer (CRC) in a large primary care database with 60,641 patients. The prediction model with both known predictive features and free-text data (with *TabTxt* AUROC: 0.823) performs statistically significantly better (p < 0.05) than the other two models with only tabular (as used nowadays) and text data, respectively (AUROC *Tab:* 0.767; *Txt*: 0.797). The specificity of the two models that use demographics and known CRC features (with specificity *Tab:* 0.321; *TabTxt:* 0.335) are higher than that of the model with only free-text (specificity *Txt*: 0.234). The *Txt* and, to a lesser degree, *TabTxt* model are well calibrated, while the *Tab* model shows slight underprediction at both tails. As expected with an outcome prevalence below 0.01, all models show much uncalibrated predictions in the extreme upper tail (top 1%). Free-text consultation notes show promising results to improve the predictive performance over established prediction models that only use structured features. Clinical future implications for our CRC use case include that such improvement may help lowering the number of referrals for suspected CRC to medical specialists.

## Introduction

The aim of this paper is to investigate whether analysis of clinical notes written by Dutch general practitioners (GP) improve performance of prediction models for the early detection of cancer. Existing solutions comprise prediction models that have the potential to identify patients at high risk, and exclude patients with low risk from further work up. In primary care practices, risk models used for cancer detection are based on traditional logistic regression research and include, among others, CAPER (for indicating symptoms), QCANCER (for indicating symptoms combined with more general data) and the Bristol-Birmingham equation (a scoring system used for identifying colorectal cancer)^1–3^; these models use only structured features (or: variables, predictors), representing for example age, sex or symptoms like ‘ICPC-D18:change in bowel habits’^1,4,5^. The scope of improvement is that currently unknown signals for earlier detection of cancer are not identified in general practice, but might be present in the records of general practitioners (GPs). In the Netherlands patients are enlisted for many years with the same primary care practice, and free-text notes of the practice do not only contain coded information about the patient, but also information about his or her family, (medical) history, consultation notes and psychological and social wellbeing. Since GP notes are usually collected over several years, they may hold earlier predictive information.

The problem that we address concerns colorectal cancer (CRC), which is, worldwide, the third most common cancer with over 1.8 million new cases yearly, and 881,000 deaths estimated in 2018^6^. In the last six years, referrals for suspected CRC have much increased, e.g., in the UK alone by 78%, with a similar growth in demand for endoscopies. Nowadays, roughly half of the referrals are made by GPs because of the presented symptoms, and the other half because of the population screening that was introduced in 2014. Research showed that GPs referred three quarter of patients within two weeks after presentation of indicating symptoms, but still more than half of the patients suffered from disseminated disease^7^. The main reason for a late referral appeared to be a low suspicion of cancer. Moreover, because CRC has notoriously difficult symptoms that overlap with other less severe diseases, most referred patients turn out not to have CRC^8^. Not only for CRC but for cancer in general, current methods for early detection did not make much progress, and about half of the patients were referred with a cancer that had already metastasized. This means there is an increasing need for improved methods for detection and selection of patients^9^.

National screening programmes (in The Netherlands done bi-annually in patients between 55 and 75 years of age) aim at identifying not only cancer, but also pre-cancerous lesions. However, the positive predictive value for cancer in case of a positive Faecal Immunochemical Test (FIT, which is the screening test) in the Netherlands is just below 5% with the used cut-off of the FIT, and still half of the patients with cancer are missed. For those patients and for patients, not included in the screening programm, early detection by the general practitioner is still necessary. Our research is aimed at clinical identification of patients who are at risk and who are not screened as positive (because they are not included in the program, refused screening or had a negative screening test).

Our hypothesis is that the general practitioner’s (GP) free-text consultation notes hold useful predictive information, unknown until now. We therefore assess the potential added performance that primary care free-text in Dutch can provide on its own and on top of the currently known predictive features using the embeddings approach.

The main contribution of our study is to demonstrate if prediction models can be improved by using free text through automatically learning predictive features in the form of word embeddings and their aggregation to represent the whole patients’ history. Earlier work used a similar approach, but on secondary care notes such as hospital discharge summaries^10^. GP notes are rather short, quite general, and cover a long time period, they overuse abbreviations and acronyms, and contain many typographical errors.

Our approach to assess the added value of free-text in prediction is to develop, evaluate, and compare three clinical prediction models: a baseline logistic regression model based on known predictive features; an embedding-based model based on free-text; and a model based on both structured features and free-text together. Both models based on free-text first transform words to embeddings and then average them to construct a patient representation. For model development, we excluded patient data up to 5 months before a diagnosis was made. We measure performance in terms of discrimination, calibration and accuracy of predicted probabilities. We investigate the prediction models on the HAGnet database of the Academic Medical Centre of Amsterdam, containing Dutch free-text notes from a group of primary care practices.

## Results

### Patient characteristics

**Figure 1:**
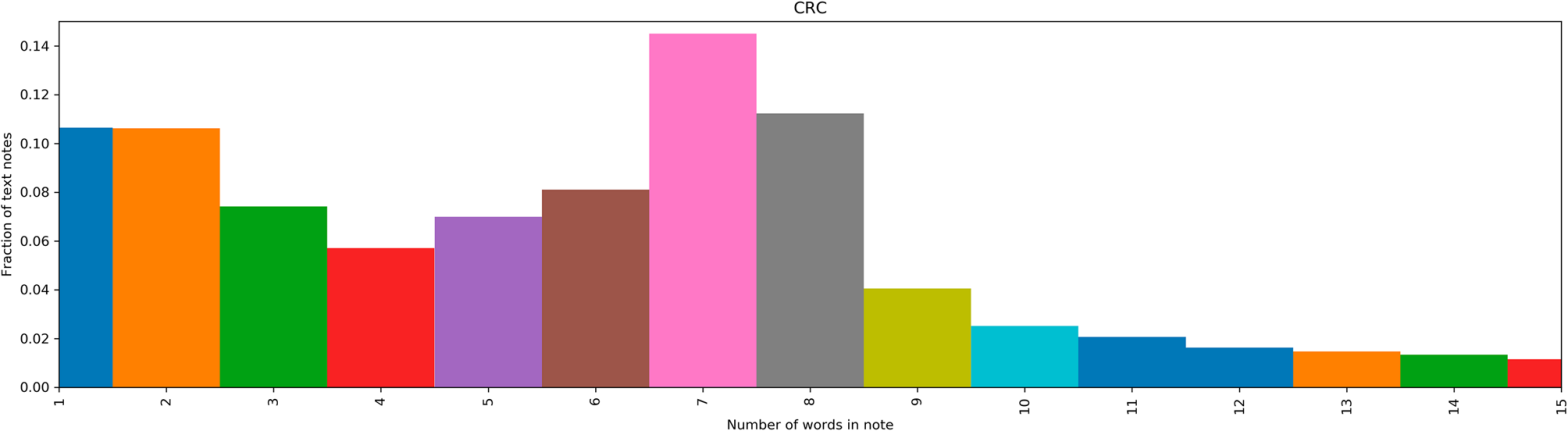
Distribution of word counts of GP consultation notes of patients with CRC.

**Figure 2:**
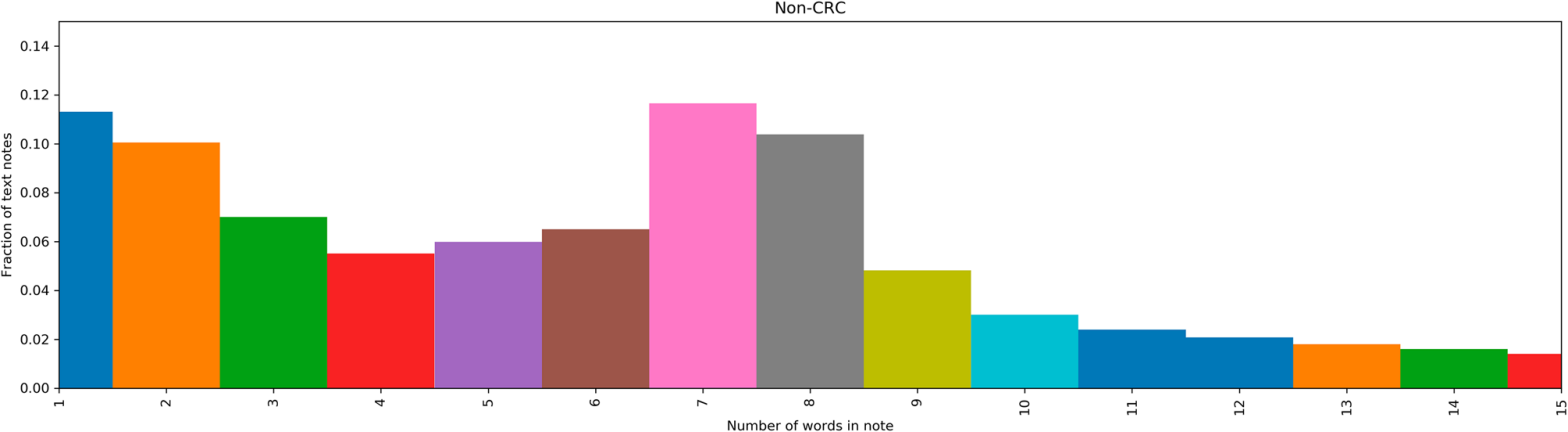
Distribution of word counts of GP consultation notes of patients without CRC.

Table 1 describes the patient characteristics of our study population and statistics on the included consultation notes. Figures 1 and 2 show the distribution of length (number of words) among the used consultation notes for patients with and without CRC, respectively. In comparison with previous findings from Dutch primary care notes^11^, our distributions are flatter (i.e., lower fractions), have longer tails (up to 15 words) and have a higher most frequent number of words (most of our notes have 7 words (fraction of text notes = 0.12). The prevalence of CRC is similar to reported rates from other observational studies on CRC with GP data in The Netherlands^10,11^ and in line with published rates by the Dutch Cancer Registry (http://www.cijfersoverkanker.nl/).

**Table 1:** Baseline characteristics of the patient population.

Category	Non-CRC	CRC
Total patients N (%)	60,100 (99.11%)	541 (0.89%)
Gender female	33,087 (55.05%)	280 (51.76%)

Age	n (%)	n (%)
[30–60)	41,242 (68.62%)	127 (23.48%)
[60–70)	8342 (13.88%)	135 (24.95%)
[70–80)	4831 (8.04%)	139 (25.69%)
[80–)	5685 (9.46%)	140 (25.88%)
Total consult notes	3,556,133	41,339

	Mean (sd)	Mean (sd)
# Per patient	59 (69)	76 (90)

### Performance measures

**Figure 3:**
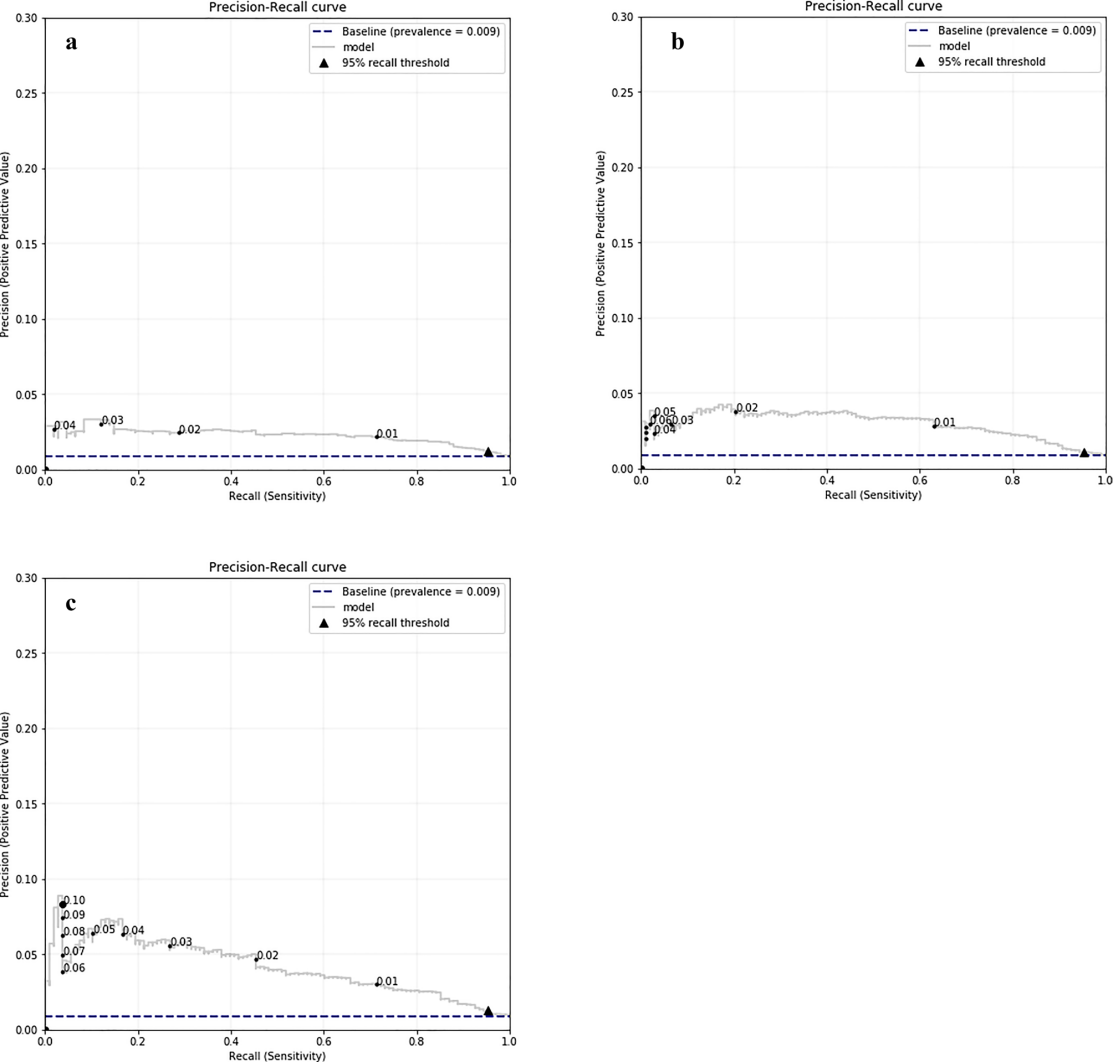
(**a**) Precision-recall curves for Tab model. Labelled dots on the curve indicate decision thresholds for the prediction model. (**b**) Precision-recall curves for Txt model. Labelled dots on the curve indicate decision thresholds for the prediction model. (**c**) Precision-recall curves for TabTxt model. Labelled dots on the curve indicate decision thresholds for the prediction model.

**Figure 4:**
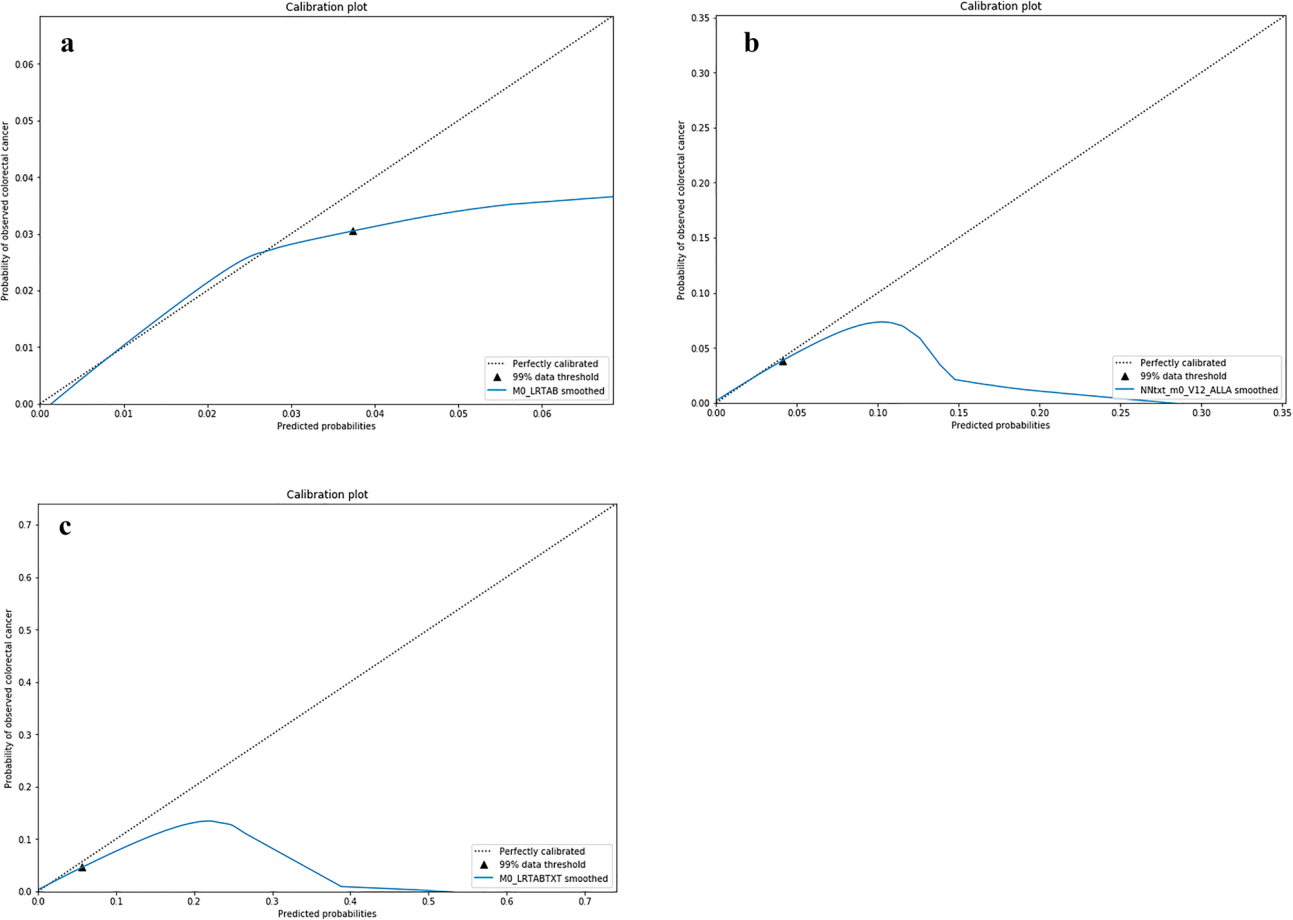
(**a**) Calibration curves for Tab model, where 99% of the predictions reside below the shown threshold. (**b**) Calibration curves for Txt model, where 99% of the predictions reside below the shown threshold. (**c**) Calibration curves for TabTxt model, where 99% of the predictions reside below the shown threshold.

Table 2 shows the Area-Under-ROC Curve; the Area-Under-PRC Curve; specificity (True Negative Rate) as achieved on the test set, along with the 95% confidence interval (CI); and Brier scores. Recall that for determining specificity, we have set the decision threshold of the model to provide a minimum sensitivity (TP/(TP + FN)) of 0.95 for CRC^4^. Figure 3 shows the precision-recall curves (PRC) of the models. For illustration purposes, we have included the decision thresholds on the curves (indicated with dots labelled with the thresholds). The numbers appearing near the dots on the lines depicted in Fig. 3a–c, denote the various decision probability thresholds, above which a diagnosis of CRC is declared. For example, for a decision threshold of 0.02 the PPV of TAB (Fig. 3a) is about 2.5%, with sensitivity of 30%. In contrast, the PPV of TabTxt for this decision threshold is about 5% with sensitivity of about 46%. This shows a marked improvement in the PPV (from 2.5 to 5%). Figure 4 shows the calibration curves for each model. The curves are smoothed using LOWESS (Locally Weighted Scatterplot Smoothing). For illustration purposes, we have indicated in the plot the 99 percentile.

**Table 2:** Median as achieved on the bootstrap samples.

Model	AUROC (95% CI)	AUPRC (95% CI)	Specificity (95% CI)	Brier score (95% CI)
*Tab*	0.767 (0.723–0.804)	0.023 (0.017–0.030)	0.321 (0.078–0.466)	**0.0087 **(0.0072–0.0104)
*Txt*	0.797 (0.752–0.837)	0.029 (0.022–0.039)	0.234 (0.139–0.515)	0.0088 (0.0074–0.0105)
*TabTxt*	**0.823 **(0.777–0.861)	**0.041 **(0.029–0.056)	**0.335 **(0.161–0.539)	0.0089 (0.0074–0.0105)

Significant values are in bold.

95% Confidence intervals from 2.5th and 97.5th percentiles. Note that for specificity, the decision threshold was set so that sensitivity was at least 0.95.

### Analysis

The TabTxt-model (with age, gender, selected structured features from CAPER and Q-cancer and free-text) performs statistically significantly better than both other models: the 95% interval (2.5th to 97.5th percentiles) of the AUROC differences between TabTxt and the Tab was (0.0271–0.0857), and between TabTxt and the Txt was (0.0031–0.0509). Both of these difference intervals indicate statistically significant differences (p < 0.05). AUPRC of all models is low, and we see that the positive predictive value (precision) rapidly decreases. Still, the positive predictive value of the text models is higher than that of the Tab model on almost all decision thresholds. Brier scores of all models are below 0.01. These Brier scores would imply high accuracy, but this is mainly a resultant of having a highly imbalanced dataset (i.e., low CRC prevalence). Brier scores for non-informative baselines are 0.0087 (0.0073–0.0105) (for prevalence) and 0.0088 (0.0074–0.0106) (for no cancer). Differences in Brier score between the TabTxt and the others were too small to consider (BSS below about 0.05 for all). For the vast majority of predictions (up until the 99 percentile), the Txt and, to a lesser degree, TabTxt model show good calibration, while the Tab model shows slight underprediction at both tails. As expected with an outcome prevalence below 0.01, all models show fairly uncalibrated predictions in the extreme upper tail (top 1%). Specificity (true negative rate, TNR) of the two models that use demographics and known CRC features (specificity Tab: 0.321; TabTxt: 0.335) are higher than that of the model with only free-text (specificity Txt: 0.234).

## Discussion

In this study we set out to investigate the predictive performance of embeddings for free-text in prediction models for colorectal cancer in primary care. Our main finding is that word embeddings for free-text did indeed improve predictive performance: adding word embeddings to established structured features resulted in a statistically significant increase in the AUROC (0.823 vs 0.767). There is no statistically significant difference between models that only use text and models that only use structured features. We observe that specificity improved in the models using structured features, while calibration improved in the models using text. Moreover, we see more than a 40% increase in AUPRC (0.041 vs 0.029 or 0.023) when combining text and structured features in one model.

Analyses of medical texts have been receiving increased attention in recent years, most notably using conventional natural language processing (NLP) techniques like entity extraction and enrichment of text with term systems^11–13^; topic modelling and extraction of clinical concepts^14^; and unsupervised text representations^15,16^. The studies used routinely collected care data, but only three studies use primary care data^11–13^. While the studies compare different prediction models and some report on internal validation (using e.g. cross validation), none has performed (or report on) statistical significance tests on the added performance value of text in the different models. The related work is briefly summarised in Table 3.

**Table 3:** Overview of related work.

Study	Data type	NLP methods	Analysis	Validation	Language
^15^	Routinely collected	Embeddings	Neural network	Internal: train/test	English
^16^	Routinely collected	Embeddings	Neural network	Internal: train/test	English
^11^	Primary care	Bag of words Topic modeling UMLS	Logistic regression	Internal: cross validation	Dutch
^12^	Primary care	Bag of words Topic modeling UMLS	Logistic regression	Internal: cross validation	Dutch
^13^	Primary care	Bag of words Topic modeling UMLS	Recurrent neural networks	Internal: cross validation	Dutch
^14^	Routinely collected	Topic modeling Concept extraction	Neural networks	Internal: train/test	English

We used actual routinely collected primary care data for a relevant clinical outcome (detection of cancer). This is the first study that uses a word embeddings approach for non-English (here: Dutch) text in primary care. We incorporate recent well-performing embedding approaches and enrich those with structured features. We used multiple performance measures including calibration curves and Brier scores (accuracy of predicted probabilities). Our approach based on aggregating word embeddings and tabular features is systematic, modular, and cheap to compute and integrates multiple types of data, which renders this approach flexible. Finally, we included a statistical comparison with a baseline structured model representing the established current models in primary care practices, i.e., Bristol-Birmingham, QCANCER and CAPER^1,3,5^. Our choice for using word embeddings without other contextual cues, like order of words in the text or external ontologies, is meant to understand the isolated incremental added value of words themselves.

A strong point of our study is that we excluded patient data up to 5 months before a diagnosis was made. Still, a limitation of our study is that the exact moment of referral is often not registered in primary care (although this is improving), which potentially leads to considering the wrong moments of diagnosis in our study^17^. Likewise, the models that only use ICPC codes may miss information that was actually observed by the GP, but which were recorded in the notes rather than in the codes. In terms of the prediction model, we did not include temporal information (dates, order) of the consultations. However, since our goal is to investigate the potential added value of words, adding such temporal information may have given the text-based models an unfair advantage. Regarding the evaluation strategy, applying bootstrapping to the whole dataset (instead of only the test set) would account for variability in choices of the training, validation, and test sets but would be extremely computationally intensive in our case and not scale to deeper language models. Concerning validation of the experimental results, external validation is needed to show how generalizable the model is to other settings. Doing so requires a similar enough but also distinct dataset that was not available to us. Still, we did perform (1) extensive internal validation, i.e., we evaluated the trained models on bootstrap samples of the test set, and (2) validation of our text models compared to other (non-text) models for early cancer diagnosis models, i.e., our *Tab*-model only uses patient data that CAPER, Bristol-Birmingham, and QCANCER use. Finally, for real world application, error analysis should be performed, including model interpretability. Such interpretability could for example be associating the strength of words on the outcome (which requires intensive computational analysis), with which we can then see which words in the text indicated the presence of CRC. Still, we note that there is no guarantee that a patient with higher risk would correspond to separate suspicious words. It is the amalgamation of implicit cues that results in the prediction and not discrete representations of words.

Although the model is yet far from being ready for clinical practice, its foreseen future application entails providing a risk estimate for CRC at each visit of the patient to the GP. The idea is that although the timing of the CRC diagnosis is unknown, once the visit would fall close to 5 months prior to that diagnosis, then the predictive performance should be similar to the one reported in this paper.

For researchers and developers of prediction models and decision support systems, we demonstrate the potential of using free-text from real-world primary care medical documentation in prediction. In terms of extending the current state of knowledge in the field, we add to the incremental evidence on the suitability of machine learning for harnessing the information residing in medical free-text. Such models are relevant for (improving) decision support systems as medical decision aids in the GP consultation room, potentially improving detection, while possibly reducing the amount of negative endoscopy referrals. Concerning the referrals for suspected CRC to medical specialists, we see that the improved specificity of the model with both structured features and text can help temper the increasing referral rate. For future application of the developed prediction model, the foreseen usage is that the model provides a prognostic risk score for CRC, months ahead of a potential diagnosis. Lastly, we emphasize that the developed and validated models in our research are *prediction* models of yet existing diseases. Therefore, these models can not be interpreted causally, nor do these provide predictions of future disease (like cardiovascular disease in patients with hypertension) which is imperative for further clinical implementation of our work. Our models may help identify cases more precisely and at an earlier stage.

Our findings suggest a need for further research, for which we do five recommendations. Firstly, linkage to a cancer registry will increase the reliability of diagnoses. Secondly, incorporating more or better-informed structured features related to BBE, CAPER and/or QCANCER (instead of the ICPC codes as we use now), as well as investigating more context (taking order of events into consideration, and including knowledge graphs) will likely improve predictive performance further. Thirdly, more data from multiple centres can increase model performance and enable external validation. Fourthly, interpretability of the model is of great importance for the acceptance of such models in decision support tools and will allow for improved error analysis. Finally, cancer development can span a number of years (for colorectal cancer up to 10 years), therefore a longitudinal analysis (f.e. comparison of annual data) could provide prediction curves assisting clinical decision making even further.

Our study shows that medical text notes can be leveraged by machine learning for prediction tasks. Specifically, for early cancer detection in primary care, free text provides added predictive value. For the clinical practice, improved prediction models can speed up and reduce referrals for suspected cancer. These contributions add to the current state of the art of risk models, since common accepted risk models used for colorectal cancer use only structured features. Besides, despite that some recent risk models use free-text, these are still used for only the English language and/or rely on external sources such as medical terminology reference lists.

## Methods

### Study design and the developed prediction models

We developed three models on data of a retrospective observational cohort. All models output a probability based on logistic regression (LR), either directly or equivalently via a node with sigmoid activation function. These models are:

1. *Tab*-model: our baseline model is an LR model combining tabular (hence the model name) features from established primary care risk models, i.e., Bristol-Birmingham, QCANCER and CAPER^1,3,5^. The features include: age (normalised), gender (three categories) and the normalised count of the ICPC codes that correspond to Constipation, Diarrhoea, Changed Bowel Habit, Abdominal Pain, Abdominal Bloating, Rectal Blood Loss, Weight Loss, Loss of Appetite, Anaemia (2) and Thrombophlebitis: D12, D11, D18, D01, D25, D16, T08, T03, B80, B82 and K94. We do not use the reported coefficients from the original papers, but instead re-train the model on our dataset.
2. *Txt*-model: a model using free-text notes in which word embeddings are averaged to represent a patient embedding (a vector). The patient embedding is fed to a logistic regression model.
3. *TabTxt*-model: a model combining the features of the first model (*Tab*) and the patient embeddings from the second model (*Txt*) and outputting a probability of CRC using logistic regression.

Further details on the *Txt* and *TabTxt* models are provided below, including model development and the used learning algorithms.

### Database and population

We used the AmsterdamUMC primary care HAGnet Electronic Medical Record database, an observational cohort of a network of six primary care practices with 40,000 current patients, and in total over 150,000 patient years for a period of over 20 years (1995–2015) containing de-identified patients information, episodes and problem lists, lab, medications, diagnosis (using the ICPC coding system), referrals, and notes written in free-text in Dutch.

### Patient inclusion

All patients in the database that are over 30 years old were included, except those without any data on the required features nor free-text in the selected time period. Per patient, CRC diagnosis was identified and labelled by the first ICPC code starting with D75 (Colon / rectum malignancy) in their record. As input data for each patient, we used two years of data recorded prior to five months before the date of registering this diagnosis, as advised by clinical judgement to prevent suspicion bias. For patients not diagnosed with CRC, we applied the same time window (two years data), up to five months before their last visit to the GP.

### Data extraction and pre-processing

The following data were extracted per patient: demographics (age, gender), consultation notes, and ICPC codes for the consultations. An example of a note in Dutch is: “Lage rugklachten bij vastzitten cl. Advies trainen met hartslameter. Loop adviezen. Kan zo een 30 minhardlopen. Niet meer depressief”, which translates to (including similar language errors): “Low backpain when stuck cl. Training advice with heart ate monitor. Walk advices. Can runfor 30 min. Not depressed anymore”. Minimal syntactic pre-processing was performed on these notes to keep potential important signals in the text: we only replaced rare tokens (i.e., tokens not equal to a–Z, 0–9, +, −, / and \) with spaces, stripped excess empty spaces and lowercased the words.

### Model development

**Figure 5:**
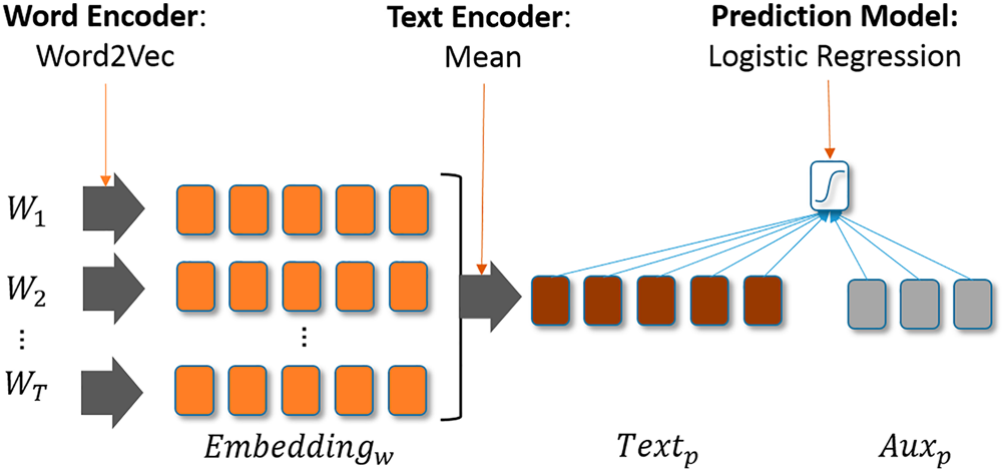
Model architecture of the used prediction models (left: text encoding; right: prediction model).

The prediction model architecture used for Txt and TabTxt (with free-text) is shown in Fig. 5 and the used learning algorithms are described in detail in the following section. We split the data randomly in stratified training (60% to train models), validation (20% for hyperparameter tuning) and testing (20% for final evaluation) sets.

### Learning algorithms

Word embedding models have been used in recent years on very large datasets (e.g., Wikipedia) in order to facilitate down-stream tasks like prediction^18,19^. The general outline of the word embedding approach is that each word in a corpus is represented by a numeric vector in a vector space of typically several hundred dimensions. During the training of a word embedding model, the word vectors are positioned (“embedded”) in the vector space where words that share a common context (taken as being semantically similar) are located “close” to each other in the vector space. For example, heart, cor, and ventricle are placed close to each other (e.g. using the Cosine similarity measure). Our prediction models (Fig. 5) are based on the word embedding models^15,16^ and consist of two parts: first, the text processing part (i.e., the word and text encoders) aggregates words into patient vectors (by averaging the word embeddings), similar to the “embed-and-aggregate” method, which is shown to perform on par with the more involved deep learning techniques like recurrent neural networks (RNNs)^15^ and the Neural Bag-of-Words approach^15,20^. Specifically, with very small target task training set sizes, the approach to learn embeddings offline (i.e., independent of the prediction task) is shown to learn representations that yield significant more accurate models; additionally, more aggressive dimensionality reduction often benefit tasks with very small training sets^15,21^. The second part trains a prediction model; possibly using extra structured features (indicated with Aux in Fig. 5) for the patient.

#### Text representations as embeddings

We process the corpus (all free-text notes in the training set plus the free-text notes of patients under 30 years old, but which are not used in the downstream task) in order to transform text to features used in the subsequent prediction models. In particular, these features are based on (aggregates of) word embeddings^18,19^. We also apply word bigrams to get embeddings for frequent bigrams (e.g. ‘colorectal cancer’) instead of just single words^19^. As shown in Fig. 5, words (${W}_{T}$) are embedded by the Word Encoder (this is the Word2Vec skip-gram trained on our corpus) into a d-dimensional (d = 300 in our case) vector. Subsequently, all embeddings are aggregated per patient, creating a single text embedding via a Text Encoder. We used the mean for aggregation, as we found it to perform better than max, min or sum on the validation set (i.e. the data set on which we tune our hyperparameters). We do not update the embeddings in the downstream prediction task, i.e. they are learned offline, as this performed better on the validation set (by keeping the number of trainable parameters low). Our approach may lose information about the order of words, but seems to work well regardless and is cheap to compute^15,22^. Once aggregated, we have one text embedding per patient p (${Text}_{p}$), representing all textual notes for this patient in a small dense fixed-size vector.

#### Prediction model

The text embedding (${Text}_{p}$) resulting from free-text processing can be fed into the (logistic regression) prediction model, together with structured input Aux_p (e.g. age and gender) for patient p.

### Hyperparameter setting

Supplementary Table S1 shows the hyperparameter settings for all models. For all prediction models, we minimise binary cross entropy loss with the Adam optimizer with early stopping on the validation set with a maximum number of 300 epochs, with a batch size of 64^23^. The embedding model is trained using the following parameters for Word2Vec: use skip-gram, embedding dimension of 300, window size of 5, using negative sampling (k = 5), threshold of 0.0001 frequency for downsampling words (i.e. more frequent words are downsampled) and a (lower) minimum word count of 3, to keep more rare misspelling variants. Note that subsampling is performed to deal with the imbalance between rare and frequent words. Frequent words (e.g., ‘the’, ‘a, et cetera) usually provide less information than rare words. We perform down-sampling where words are discarded with a probability inverse to their frequency: this strategy “aggressively subsamples words whose frequency is greater than a threshold t (here: 0.00001) while preserving the ranking of the frequencies”^18^. While down-sampling is the prevailing strategy to discard frequent words, over-sampling to boost rare words may be considered as well^24^.

### Performance measures

We measured predictive performance, in terms of discrimination, with the area under the receiver-operator curve (AUROC), and the balance between the positive predictive value and sensitivity with the area under the precision-recall curve (AUPRC). We compared the three models on their respective AUROC values. We report specificity of each model at the threshold that results in a sensitivity of 0.95. This in order to have a fair comparison between the model performances when not missing more than 5% cancer cases, which is a medically relevant score. A sensitivity of 0.95 means that although a relatively large number of patients might need to get more attention from the GP based on their risk (for example being seen again by the GP in a follow-up visit, or directly referred) these patients are much better selected than subjecting a huge number of patients for screening. As such, our model provides a rational approach to decide on those needing more attention. We included calibration curves. These indicate how close the predicted probabilities are to the true rate of the observed event across the full probability range. We use the Brier score to measure (in)accuracy of the predicted probabilities, summarising deviations between actual and predicted outcomes at the patient level^25,26^. Brier score is the mean squared error of predicted outcome, where lower Brier scores indicate better accuracy. We use Brier Skill Score (BSS) to measure the improvement of the models over non-informative baseline prediction models: one model that predicts the prevalence baseline (p(x) = 0.0089) to all patients, and the second is the majority-rule model that predicts no cancer for everyone (p(x) = 0). A BSS score of 1 indicated maximum improvement, 0 is no improvement and a negative value indicates worse performance.

### Evaluation strategy

We evaluated the trained models on 1000 bootstrap samples of the test set. We recorded the median AUROC of the bootstrap results as the primary result, while using the percentile bootstrap method to provide a 95% confidence interval from the 2.5th and 97.5th percentiles. We also calculated statistical significance based on the percentile bootstrap method on the differences in AUROC between any two models: we took the 2.5th and 97.5th percentiles and inspect whether 0 is included in the remaining 95% interval; if it is not, then there is a statistically significant difference between these results with p < 0.05.

### Statistical analysis

All analyses were performed using Python v3 with Gensim (word embeddings) and Keras (prediction model) modules. Hardware included a consumer laptop (for data preprocessing: pseudonymization and embeddings) and a cloud virtual machine (for prediction algorithms and evaluation) without a GPU.

### Ethical approval

The records in the used dataset with retrospective, routinely collected observational data were de-identified. As such, this research was exempted by the Medical-Ethical Research board of Amsterdam UMC (Medisch Ethische Toetsingscommissie AMC; Meibergdreef 9, 1105 AZ Amsterdam; W21_197 # 21.214).

### Informed consent

Informed consent was obtained from all subjects and/or their legal guardian(s) by means of an opt-out system in case subjects objected to using their data.

### Guidelines statement

All the items in the TRIPOD guidelines reporting statement pertaining to the development of a multivariable prediction model have been considered and are addressed in this paper^27^.

## Supplementary Information

Supplementary Table S1.

## Data Availability

Data cannot be shared publicly because of patient confidentiality. Data are available from the HAG research network (contact via https://www.amc.nl/web/leren/huisartsopleiding-1/huisartsgeneeskunde/hagnet-amconderzoeksnetwerk.htm) for researchers who meet the criteria for access to confidential data.
