# Supplementary material for: Early detection of colorectal cancer by leveraging Dutch primary care consultation notes with free text embeddings
Source: Sci Rep. 2023 Jul 4;13:10760. doi: 10.1038/s41598-023-37397-2 (PMC10319709; doi:10.1038/s41598-023-37397-2)
Supplement: Supplementary file 1 — Supplementary Table S1. [file 41598_2023_37397_MOESM1_ESM.docx]

**Table S1: Hyperparameter settings**

| **Hyperparameter** | ***Tab*** | ***Txt*** | ***TabTxt*** |
| --- | --- | --- | --- |
| **Optimiser** | Adam (default settings) | Adam (default settings) | Adam (default settings) |
| **Epochs** | 158 | 83 | 297 |
| **Batch Size** | 64 | 64 | 64 |
| **Hidden Layers** | 0 | 0 | 0 |
| **Output - Nodes** | 1 | 1 | 1 |
| **Output - Activation Function** | Sigmoid | Sigmoid | Sigmoid |
| **Loss Function** | Binary Cross-Entropy | Binary Cross-Entropy | Binary Cross-Entropy |
| **Class Weights** | [ 0.5045  55.9754] | [ 0.5045 55.9754] | [ 0.5045  55.9754] |
| **Embeddings - Pretrained** | - | Yes | Yes |
| **Embeddings - Offline** | - | Yes | Yes |
| **Embeddings - Dimensions** | - | 300 | 300 |
| **Embeddings - Method** | - | Word2Vec | Word2Vec |
| **Word2Vec - Method** | - | Skip-Gram | Skip-Gram |
| **Word2Vec - Window** | - | 5 | 5 |
| **Word2Vec - Sample** | - | 0.0001 | 0.0001 |
| **Word2Vec - Min Count** | - | 3 | 3 |
| **Embeddings - Aggregation** | - | Mean | Mean |
| **Training Data – Prediction** | Train split  (60%, stratified) | Train split  (60%, stratified) | Train split  (60%, stratified) |
| **Training Data – Embeddings** | - | Train split  (60%, stratified)  + ages < 30 | Train split  (60%, stratified)  + ages < 30 |
| **Uses tabular data** | Yes | No | Yes |
| **Aux – Age** | Normalised | - | Normalised |
| **Aux – Sex** | 3 Categories | - | 3 Categories |
| **Aux – CRC code values** | Normalised counts | - | Normalised counts |
| **Aux – CRC codes** | 11 Categories | - | 11 Categories |
| **Feature Combination** | No | No | Concatenation |
